# Audiovisual Segregation in Cochlear Implant Users

**DOI:** 10.1371/journal.pone.0033113

**Published:** 2012-03-12

**Authors:** Simon Landry, Benoit A. Bacon, Jacqueline Leybaert, Jean-Pierre Gagné, François Champoux

**Affiliations:** 1 Centre de Recherche en Neuropsychologie et Cognition (CERNEC), Montréal, Québec, Canada; 2 Department of Psychology, Bishop's University, Sherbrooke, Québec, Canada; 3 Université libre de Bruxelles, Bruxelles, Belgique; 4 Centre de recherche interdisciplinaire en réadaptation du Montréal métropolitain, Institut Raymond-Dewar, École d'orthophonie et d'audiologie, Université de Montréal, Montréal, Québec, Canada; Baycrest Hospital, Canada

## Abstract

It has traditionally been assumed that cochlear implant users de facto perform atypically in audiovisual tasks. However, a recent study that combined an auditory task with visual distractors suggests that only those cochlear implant users that are not proficient at recognizing speech sounds might show abnormal audiovisual interactions. The present study aims at reinforcing this notion by investigating the audiovisual segregation abilities of cochlear implant users in a visual task with auditory distractors. Speechreading was assessed in two groups of cochlear implant users (proficient and non-proficient at sound recognition), as well as in normal controls. A visual speech recognition task (i.e. speechreading) was administered either in silence or in combination with three types of auditory distractors: i) noise ii) reverse speech sound and iii) non-altered speech sound. Cochlear implant users proficient at speech recognition performed like normal controls in all conditions, whereas non-proficient users showed significantly different audiovisual segregation patterns in both speech conditions. These results confirm that normal-like audiovisual segregation is possible in highly skilled cochlear implant users and, consequently, that proficient and non-proficient CI users cannot be lumped into a single group. This important feature must be taken into account in further studies of audiovisual interactions in cochlear implant users.

## Introduction

It has been shown numerous times that when congruent visual and auditory cues are processed together perceptual accuracy is enhanced in both normally-hearing (NH) and in hearing-impaired individuals (e.g. [1]–[13]). In contrast, several investigations have demonstrated that when incongruent visual and auditory cues are processed together, audiovisual interactions seem to occur differently in hearing-impaired individuals, compared to NH. Specifically, audiovisual perception is dominated by auditory information in the NH, whereas it is dominated by vision in hearing-impaired individuals that are using a cochlear implant (CI). For example, a number of studies that used a McGurk effect paradigm [14] have shown that CI users are able to integrate auditory and visual information adequately [12], [15], [16] but that they essentially refer to the visual cues when incongruency makes integration difficult [11], [17], [18].

Tremblay et al. [19] recently suggested that when it came to audiovisual integration, not all CI users could be grouped together. In an audiovisual fusion task, only the CI users that were unable to recognize auditory speech sounds efficiently (yet showed normal sound detection performance) were referring primarily to visual cues to process speech information. Interestingly, the results of Tremblay et al. [19] also suggest that a number of CI users, namely those who are proficient in sound recognition, can show normal-like audiovisual interactions even in situations of incongruity. This notion is strongly supported by a recent study of audiovisual segregation; namely the ability to focus on the processing of one information stream while ignoring the irrelevant and incongruent information in an audiovisual task [20]. In an auditory task with visual distractors, proficient CI users performed in a normal-like manner, while non-proficient users did not; they were in fact much more disturbed by the visual distractors that involved movement (dots, lip movements) but not, however, by color changes. To our knowledge, this remains the only study of audiovisual segregation ability in CI users.

These two investigations [19], [20] contrast with the general idea that all CI users rely more heavily on visual cues in conditions of incongruency [11], [12], [15]–[18]. More specifically, the results suggest that i) CI users can show normal-like performance in an audiovisual task, with the normal relative weight of visual and auditory cues, and ii) only the CI users that are non-proficient in highly demanding auditory tasks, such as speech identification, show abnormal, visual-oriented interactions.

Performance on audiovisual segregation tasks, however, has to be carefully assessed in order to fully confirm these conclusions. In particular, the question remains as to whether the reverse task, namely ignoring auditory distractors in a visual task, is performed differently in proficient and non-proficient CI users. The present study tackled this issue by comparing proficient CI users, non-proficient CI users and NH in a speechreading task with and without auditory distractors. In accordance with the results of Champoux et al. [20], it was hypothesized that only non-proficient users would differ from the NH. More precisely, it was hypothesized that speechreading would be affected by incongruent auditory information in NH and proficient CI, but not in non-proficient CI. The confirmation of these hypotheses would in effect also demonstrate that the results reported in Champoux et al. [20] were not due to the specificity of the task, the procedure or the stimuli used and confirm further the possibility of normal-like audiovisual interations in cochlear implant users.

## Methods

### Subjects

**Figure 1:**
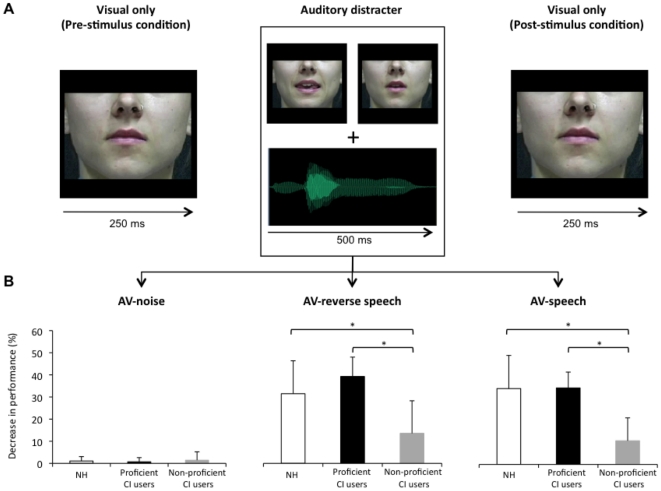
Experimental procedure and visual speech recognition performance in the three audiovisual conditions. (A) Illustration of the experimental procedure. Each visual stimulus began and ended in a static neutral position. Visual stimuli were either presented alone (baseline condition) or simultaneously with one of three types of auditory stimuli (white noise, reverse speech sounds and non-altered speech sounds). (B) Performance in the three audiovisual conditions is expressed as % decrease of performance compared to the baseline visual-only condition. In both the AV-reverse speech and AV-speech conditions, non-proficient CI users differ from both controls and proficient CI users. * : *p*<.05.

Twenty-four participants (seventeen CI users) were involved in the study. All CI users had received their implants at least one year prior to taking part in the study. The clinical profile of each participant has been described elsewhere (see [20]). All participants suffered from profound bilateral hearing loss (pure-tone detection thresholds at 80 dB HL or greater at octave frequencies ranging from 0.5 to 4 KHz) and were post-lingually deafened. The principal communication mode for all CI users was oral/lip-reading. In all participants, pure-tone detection thresholds with the CI, at octave frequencies ranging from 250 to 6000 Hz were within normal limits (30 dB HL or less). The Research Ethics Board of the Institut Raymond-Dewar approved the study and all the participants provided written informed consent. Figure 1 includes the picture of an audiologist. We confirm that this individual has seen this figure and the manuscript, and has provided written consent for publication.

### Stimuli and design

A female speaker was filmed while she pronounced 120 consonant-vowel-consonant-vowel bi-syllabic words. The production of each stimulus began and ended in a neutral, closed mouth position and total duration of the stimuli was about 500 ms. Stimuli were presented in a baseline visual-only condition or in one of three incongruent audiovisual conditions (see Figure 1A). In the first audiovisual condition (AV-noise), visual stimuli were presented together with a comfortable level of white noise. The noise was generated with Cool Edit pro software (version 1.2: Syntrillium Software Corporation, San Jose, CA). This condition served as a second baseline and no difference was expected with the visual-only condition. In the second audiovisual condition (AV-reverse speech), visual stimuli were presented with reverse-speech sounds of the bisyllabic words. In the third audiovisual condition (AV-speech), visual stimuli were presented with non-altered speech sounds of the bisyllabic words. Temporal synchrony between the visual stimulus and the auditory utterance was achieved by aligning the burst corresponding to the beginning of the test word in the auditory condition with the appearance of motion in the visual stimulus. An informal pre-evaluation confirmed that the reverse-speech and speech auditory stimuli were clearly detectable and identifiable as speech sounds by both proficient and non-proficient CI users.

### Procedure

Forty stimuli from each of the three audiovisual conditions were presented in one block of 120 trials. The order of the stimuli was randomized with Presentation software (Neurobehavioral Systems Inc., San Pablo, CA). The visual stimuli were presented on a 17″ video monitor that was positioned at the participant's eye level at a viewing distance of 114 cm. The auditory stimuli were always presented at a comfortable listening level via two loudspeakers positioned at ear level and located on each side of the video monitor. The participants were asked to look at the screen, to completely ignore what they heard and to only report what they had read on the lips of the speaker. They were clearly informed that auditory input would always be incongruent with the visual stimulus and that their task was to report the visual stimulus. An experimenter was present throughout the procedure to ensure that the participants were looking at the screen before stimulus presentation and to monitor oculomotor behavior during stimulus presentation.

The procedure used to analyze segregation abilities has been described previously (see [20]). Prior to data collection, auditory speech recognition was measured in a counterbalanced order with a list of 40 bisyllabic words. These results confirmed those obtained with the same sample by Champoux et al. [20]. Most importantly, the proficient and non-proficient groups remained stable. The performance level of three CI users in the auditory-alone condition was extremely low, as these participants were barely able to differentiate speech from non-speech sounds. Hence, the results of these participants were not considered in the data analyses. Whereas the ability to accurately identify words presented auditorily varied considerably, all the other CI users (n = 14) were able to make the distinction between speech and non-speech sounds. These CI participants were divided in two groups: Proficient (n = 7) when their auditory speech recognition performance as measured with a list of 40 bisyllabic words was above 75% and non-proficient (n = 7), when auditory speech recognition performance was below 75%. The visual-alone baseline condition was used as a reference point from which to compute the percent decrement in performance in each of the three audiovisual conditions, i.e. decrease of performance = (% score in the visual-alone condition – % score in the audiovisual condition). A t-test found no significant difference (*p*>.05) between proficient and non-proficient CI users in the ability to discriminate bisyllabic words in a congruent audio-visual or visual-alone condition.

## Results

Visual speech recognition performance in the three incongruent audiovisual conditions is shown in Figure 1B. To determine speechreading ability with or without irrelevant auditory distractors, a 3×3 mixed ANOVA with group (control, proficient CI users, non-proficient CI users) as a between-subjects factor and audiovisual condition (AV-noise, AV-reverse speech, AV-speech) as a within-subjects factor was conducted. There were main effects of condition (F(2,36) = 114.537, *p*<.001) and group (F(2,18) = 9.901, *p* = .001). The interaction between factors was also significant (F(4,36) = 10.959, *p* = .001). Post-hoc Tukey HSD tests revealed significant differences between the non-proficient group and the control group in the AV-reverse speech (*p* = .049) and AV-speech (*p* = .005) conditions. There were also significant differences between the non-proficient and the proficient group in the AV-reverse speech (*p* = .005) and AV-speech conditions (*p*<.001). Post-hoc analysis did not reveal any other differences between groups (*p*>0.05) and as such the performance of proficient CI users was never statistically different from that of the NH controls. The performance level of every CI user was examined further in the three experimental conditions. There was a significant correlation between the decrease in speechreading performance and the proficiency to use the CI in the AV-reverse speech (r = 0.686, *p* = .007) and the AV-speech (r = 0.824, *p*<.001) conditions. There were however no significant relationships (*p*>.05) between visual recognition performance and the duration of deafness, the age at onset of hearing loss or the length of experience with CI.

## Discussion

The present study aimed to investigate audiovisual segregation abilities in proficient and non-proficient CI users. Using a speechreading task and three types of auditory distractors, we showed that the presentation of auditory speech stimuli significantly impaired speechreading performance in proficient CI users, just like in NH participants, whereas speechreading performance was unaffected by auditory distractors in non-proficient CI users.

Traditionally, all CI users have been considered equal, and equally different from NH, in audiovisual tasks. In short, it is assumed that this population, although capable of normal integration, tends to rely more heavily on visual cues in conditions of incongruency (e.g. [11], [12], [15]–[18]. However, recent evidence from our laboratories [19], [20] highlights the importance of CI proficiency in audiovisual interaction outcomes. We suggested that whereas CI users that were proficient at speech recognition could perform at normal-like levels, those that were not would favor visual cues and show anomalous audiovisual integration. The results presented here therefore support two notions: *i*) that CI speech recognition proficiency is associated with audiovisual interaction outcomes in this population and *ii*) that several CI users, namely the proficient ones, can show normal-like performance on an audiovisual task.

Cross-modal reorganization has been repeatedly shown to occur in the profoundly deaf (e.g. [21]–[24]). In fact, in CI users, there is an activation of the early auditory cortex in the presence of visual stimuli and this activation is greater for those who show poor speech recognition abilities [25]. In addition, CI users display atypical low-hierarchical visual activity during speech recognition tasks [26]. This activity in the visual cortex is less marked and less consistent in naive than in rehabilitated CI users, suggesting that these visual cortex activations are due not only to deafness-induced plasticity, but also to brain reorganizations related to the functional learning of associations between visual cues and oral speech [25]. Therefore, different levels of auditory-to-visual reorganization in cochlear implanted deaf subjects could explain the varying audiovisual segregation abilities reported in the present study: greater cross-modal reorganization would lead to the overuse of visual information and consequently to a greater capacity to ignore irrelevant auditory cues.

Some other issues, however, could also explain the pattern of results observed across tasks and groups. First, the three audiovisual tasks arguably did not require the exact same attentional resources. Indeed, speech stimuli were more salient and more complex than noise stimuli and consequently, were more likely to capture attention. Some studies, moreover, suggest that children with CI could perform poorly on attentional tasks [27], [28], although performance might improve progressively with the use of a CI [29]. In our study, putative impairments of visual or auditory attentional processes have unfortunately not been evaluated. These capacities might need to be investigated further in those populations to better understand their implications in the present results.

In conclusion, our results strongly suggest that in terms of audiovisual interactions, proficient and non-proficient CI users should not be lumped into a single group. More specifically, we show that normal-like audiovisual interactions are possible in proficient users and we show that CI proficiency is associated with audiovisual interactions in CI users. CI proficiency must therefore be taken into account in further studies of audiovisual interactions in this population.

## References

[1] Perrott DR, Saberi K, Brown K, Strybel TZ (1990). Auditory psychomotor coordination and visual search performance.. Percep Psychophys.

[2] Hughes HC, Reuter-Lorenz PA, Nozawa G, Fendrich R (1994). Visual-auditory interactions in sensorimotor processing: saccades versus manual responses.. J Exp Psychol Hum Percept Perform.

[3] Frens MA, Van Opstal AJ, Van der Willigen RF (1995). Spacial and temporal factors determine auditory-visual interactions in human saccadic eye movements.. Percep Psychophys.

[4] Grant KW, Walden BE, Seitz PF (1998). Auditory-visual speech recognition by hearing-impaired subjects: consonant recognition, sentence recognition, and auditory-visual integration.. J Acoust Soc Am.

[5] McDonald JJ, Teder-Sälejärvi WA, Hillyard SA (2000). Involuntary orienting to sound improves visual perception.. Nature.

[6] Teder-Sälejärvi WA, McDonald JJ, Di Russo F, Hillyard SA (2002). An analysis of audio-visual crossmodal integration by means of event-related potential (ERP) recordings.. Brain Res Cogn Brain Res.

[7] Frassinetti F, Bolognini N, Làdavas E (2002). Enhancement of visual perception by crossmodal visuo-auditory interaction.. Exp Brain Res.

[8] Sekiyama K, Kanno I, Miura S, Sugita Y (2003). Auditory-visual speech perception examined by fMRI and PET.. Neurosci Res.

[9] Alegria J, Lechat J (2005). Phonological processing in deaf children: when lipreading and cues are incongruent.. J Deaf Stud Deaf Educ.

[10] Bolognini N, Rasi F, Làdavas E (2005). Visual localization of sounds.. Neuropsychologia.

[11] Van Wassenhove V, Grant KW, Poeppel D (2005). Visual speech speeds up the neural processing of auditory speech.. Proc Natl Acad Sci U S A.

[12] Rouger J, Lagleyre S, Fraysse B, Deneve S, Deguine O (2007). Evidence that cochlear-implanted deaf patients are better multisensory integrators.. Proc Natl Acad Sci U S A.

[13] Ross LA, Saint-Amour D, Leavitt VM, Molholm S, Javitt DC (2007). Impaired multisensory processing in schizophrenia: deficits in the visual enhancement of speech comprehension under noisy environmental conditions.. Schizophr Res.

[14] McGurk H, McDonald J (1976). Hearing lips and seeing voices.. Nature.

[15] Schorr EA, Fox NA, van Wassenhove V, Knudsen EI (2005). Auditory-visual fusion in speech perception in children with cochlear implants.. Proc Natl Acad Sci U S A.

[16] Schwartz JL (2010). A reanalysis of McGurk data suggests that audiovisual fusion in speech perception is subject-dependent.. J Acoust Soc Am.

[17] Rouger J, Fraysse B, Deguine O, Barone P (2008). McGurk effects in cochlear-implanted deaf subjects.. Brain Res.

[18] Desai S, Stickney G, Zeng FG (2008). Auditory-visual speech perception in normal-hearing and cochlear-implant listeners.. J Acoust Soc Am.

[19] Tremblay C, Champoux F, Lepore F, Théoret H (2010). Audiovisual fusion and cochlear implant proficiency.. Restor Neurol Neurosci.

[20] Champoux F, Lepore F, Gagné JP, Théoret H (2009). Visual stimuli can impair auditory processing in cochlear implant users.. Neuropsychologia.

[21] Nishimura H, Hashikawa K, Doi K, Iwaki T, Watanabe Y (1999). Sign language “heard” in the auditory cortex.. Nature.

[22] Finney EM, Fine I, Dobkins KR (2001). Visual stimuli activate auditory cortex in the deaf.. Nat Neurosci.

[23] Sadato N, Okada T, Honda M, Matsuki K, Yoshida M (2005). Cross-modal integration and plastic changes revealed by lip movement, random-dot motion and sign languages in the hearing and deaf.. Cereb Cortex.

[24] Bavelier D, Dye MW, Hauser PC (2006). Do Deaf individuals see better?. Trends Cogn Sci.

[25] Doucet ME, Bergeron F, Lassonde M, Ferron P, Lepore F (2006). Cross-modal reorganization and speech perception in cochlear implant users.. Brain.

[26] Giraud AL, Truy E, Frackowiak R (2001). Imaging plasticity in cochlear implant patients.. Audiol Neurootol.

[27] Smith LB, Quittner AL, Osberger MJ, Miyamoto R (1998). Audition and visual attention: the developmental trajectory in deaf and hearing populations.. Dev Psy.

[28] Yucel E, Derim D, Celik D (2008). The needs of hearing impaired children's parents who attend to auditory verbal therapy-counseling program.. Int J Pediatr Otorhinolaryngol.

[29] Horn DL, Davis RA, Pisoni DB, Miyamoto RT (2005). Development of visual attention skills in prelingually deaf children who use cochlear implants.. Ear Hear.

